# Species Identification of Oaks (*Quercus* L., Fagaceae) from Gene to Genome

**DOI:** 10.3390/ijms20235940

**Published:** 2019-11-26

**Authors:** Xinbo Pang, Hongshan Liu, Suran Wu, Yangchen Yuan, Haijun Li, Junsheng Dong, Zhaohua Liu, Chuanzhi An, Zhihai Su, Bin Li

**Affiliations:** 1 Research Institute of Forestry, Chinese Academy of Forestry, Beijing 100091, China; pxb15633717296@163.com; 2 Administration Bureau of Hongyashan State Owned Forest Farm in Yixian County, Yixian 074200, China; lhs13803120634@163.com (H.L.); wsr15830855155@163.com (S.W.); 18730272192@163.com (Y.Y.); lhj13831238335@163.com (H.L.); 13930286026@163.com (J.D.); yyc16603261128@163.com (Z.L.); Anchuanzhi2002@163.com (C.A.); szh13833020580@163.com (Z.S.); 3 State Key Laboratory of Tree Genetics and Breeding, Chinese Academy of Forestry, Beijing 100091, China; 4 Key Laboratory of Tree Breeding and Cultivation of State Forestry Administration, Chinese Academy of Forestry, Beijing 100091, China

**Keywords:** oak species identification, chloroplast genome, *Quercus*, mutation hotspots

## Abstract

Species identification of oaks (*Quercus*) is always a challenge because many species exhibit variable phenotypes that overlap with other species. Oaks are notorious for interspecific hybridization and introgression, and complex speciation patterns involving incomplete lineage sorting. Therefore, accurately identifying *Quercus* species barcodes has been unsuccessful. In this study, we used chloroplast genome sequence data to identify molecular markers for oak species identification. Using next generation sequencing methods, we sequenced 14 chloroplast genomes of *Quercus* species in this study and added 10 additional chloroplast genome sequences from GenBank to develop a DNA barcode for oaks. Chloroplast genome sequence divergence was low. We identified four mutation hotspots as candidate *Quercus* DNA barcodes; two intergenic regions (*matK-trnK-rps16* and *trnR-atpA*) were located in the large single copy region, and two coding regions (*ndhF* and *ycf1b*) were located in the small single copy region. The standard plant DNA barcode (*rbcL* and *matK*) had lower variability than that of the newly identified markers. Our data provide complete chloroplast genome sequences that improve the phylogenetic resolution and species level discrimination of *Quercus*. This study demonstrates that the complete chloroplast genome can substantially increase species discriminatory power and resolve phylogenetic relationships in plants.

## 1. Introduction

DNA barcoding has recently emerged as a new molecular tool for species identification [1]. A DNA barcode is a short, standardized DNA region normally employed for species identification. The mitochondrial gene cytochrome oxidase 1 (*COI*) is an effective and reliable standard animal DNA barcode for species identification [1]. Over the past 10 years, plant DNA barcode researchers have been evaluating the proposed barcode segments of plants. Previously proposed barcode segments exist primarily in chloroplast genomes that are relatively stable, single-copy, and easy to amplify. These proposed barcodes are *matK*, *rbcL*, *ropC1*, and *rpoB* in the coding region, and *atpF-H*, *trnL-F*, *trnH-psbA*, and *psbK-I* in the non-coding region [2]. At the third DNA barcode conference held in Mexico City in 2009, the majority of the Consortium for the Barcode of Life (CBOL) Plant Working Group preferred to recommend a core-barcode combination consisting of portions of two plastid coding regions, *rbcL* and *matK*, which are supplemented with additional markers (such as *trnH-psbA* and internal transcribed spacers [ITS]) as required. In 2011, the China Plant BOL Group suggested using ITS as the plant DNA barcode [3]. However, increasing numbers of studies show that core-barcodes remain problematic, especially in recently diverged and rapidly radiated taxa [4,5,6].

With the development of next-generation sequencing (NGS), the number of sequenced chloroplast genomes has increased rapidly, making it possible to generate chloroplast genome data to extend the concept of DNA barcoding for plant species identification [6,7,8,9]. The DNA barcoding approaches for species identification has extended from gene to genome, promptly extending phylogeny analysis from gene-based phylogenetics to phylogenomics. Chloroplast genome sequences are a primary source of data for inferring plant phylogenies and DNA barcoding because of their conserved gene content and genome structure, low nucleotide substitution mutation rates, usually uni-parental inheritance, and the low cost of generating whole chloroplast genomes with high throughput sequencing. Using chloroplast genome data, longstanding controversies at various taxonomic levels have been resolved [10,11,12], suggesting its power in resolving evolutionary relationships. However, challenges still exist in establishing phylogeny relationships and discrimination of closely related, recently divergent, hybridized, or introgressed lineages such as the oak group.

Oaks (*Quercus* L., Fagaceae) comprise approximately 400–500 species that are widespread throughout the temperate zones of the Northern Hemisphere; they are dominant, diverse forest and savannah angiosperm trees and shrubs belonging to a taxonomically complex group. The taxonomy of oak species remains controversial and incomplete, owing to the overlapping variation of individuals and population produced by ecological adaptation and differential reproductive isolation. A series of phylogenetic and DNA barcoding studies have mainly used several chloroplast DNA markers [13,14] such as *rbcL*, *rpoC1*, *trnH-psbA*, *matK*, *ycf3-trnS*, *ycf1*, and the nuclear ribosomal DNA ITS [4,15,16,17]. These studies focused only on regional flora, and those markers revealed low sequence divergence leading to lower discrimination success [4,18]. Yang et al. [13] compared two closely related species (*Quercus rubra* and *Castanea mollissima*) by exploring nine highly variable chloroplast DNA markers for species identification. However, the results showed a very low discrimination success rate using a single marker and all their combinations. On the other hand, oaks are notorious for interspecific hybridization and introgression, as well as complex speciation patterns involving incomplete lineage sorting [19,20,21], which have possible negative effects for barcoding and phylogeny of the species-rich *Quercus* genus [4].

In this study, we sequenced the complete chloroplast genome of 14 *Quercus* species and combined the previously reported chloroplast genomes of 10 other *Quercus* species in order to provide a comparative analysis. The study aimed to (1) investigate the genome structure, gene order, and gene content of the whole chloroplast genome of multiple *Quercus* species; (2) test whether chloroplast genome data yielded sufficient variation to construct a well-supported phylogeny of *Quercus* species; and (3) determine if multiple variable markers or whole chloroplast genome data can be successfully used for oaks species identification.

## 2. Results

### 2.1. General Features of the Quercus Chloroplast Genome

Using the Illumina HiSeq X Ten system, 14 *Quercus* species were sequenced to produce 9,910,273–16,862,000 paired-end raw reads (150 bp average read length), with an average sequencing depth of 162× to 480× (Table S1). To validate the accuracy of the assembled chloroplast genome, we carried out Sanger sequencing of PCR amplicons spanning the junction regions (LSC/IRA, LSC/IRB, SSC/IRA, and SSC/IRB). The 14 *Quercus* chloroplast genome sequences were deposited in GenBank (accession numbers MK105451–MK105453, MK105456–MK105464, and MK105466-MK105467).

**Figure 1:**
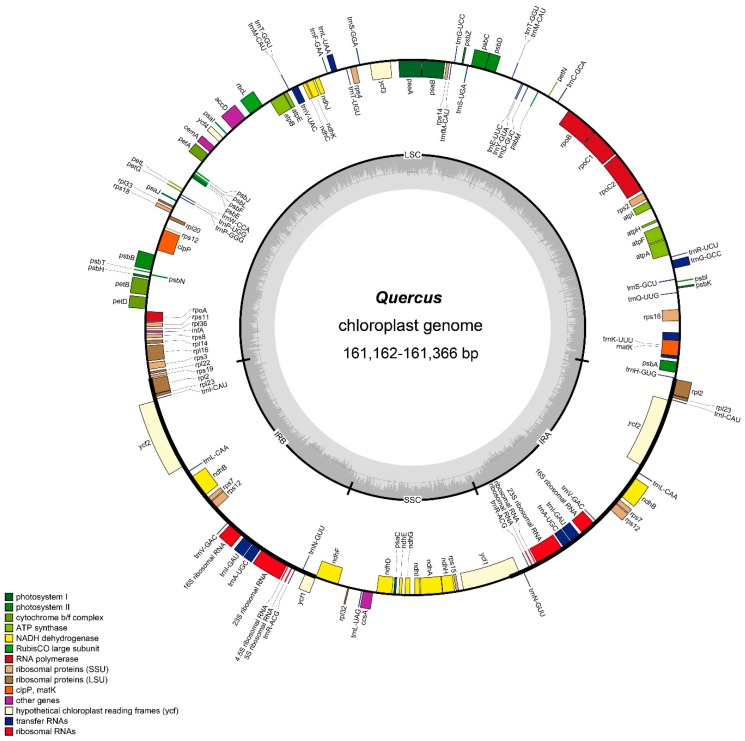
Gene map of *Quercus* chloroplast genome. Genes drawn within the circle are transcribed clockwise; genes drawn outside are transcribed counterclockwise. Genes in different functional groups are shown in different colors. Dark bold lines indicate the extent of the inverted repeats (IRa and IRb) that separate the genomes into small single-copy (SSC) and large single-copy (LSC) regions.

The total chloroplast genome sequence lengths of 14 *Quercus* species ranged from 161,132 bp (*Q. phillyraeoides*) to 161,366 bp (*Q. rubra*). These genomes displayed typical circular quadripartite structure consisting of a pair of IR regions (25,817–25,870 bp) separated by an LSC region (90,363–90,624 bp) and an SSC region (18,946–19,073 bp) (Figure 1). The overall GC content was absolutely identical (36.8%; Table 1) across all plastomes, but was clearly higher in the IR region (42.8%) than in the other regions (LSC 34.7%; SSC 30.9%), possibly because of the high GC content of the rRNA that was located in the IR regions. All plastomes possessed 113 unique genes, including 79 protein-coding genes, 30 tRNA genes, and 4 rRNA genes. Among the unique genes, 15 genes contained one intron, and two genes contained two introns.

The chloroplast genome results showed that all 14 *Quercus* plastomes were remarkably similar in terms of size, genes, and genome structures. The LSC/IR and IR/SSC boundaries were conserved. *Rps19* was located in the LSC near the LSC/IRb, and *trnH-GUG* was located in the LSC near the IRa/LSC border. Additionally, the location of the SSC/IRa junction was within the coding region of the *ycf1* gene.

### 2.2. Phylogenetic Analyses

**Figure 2:**
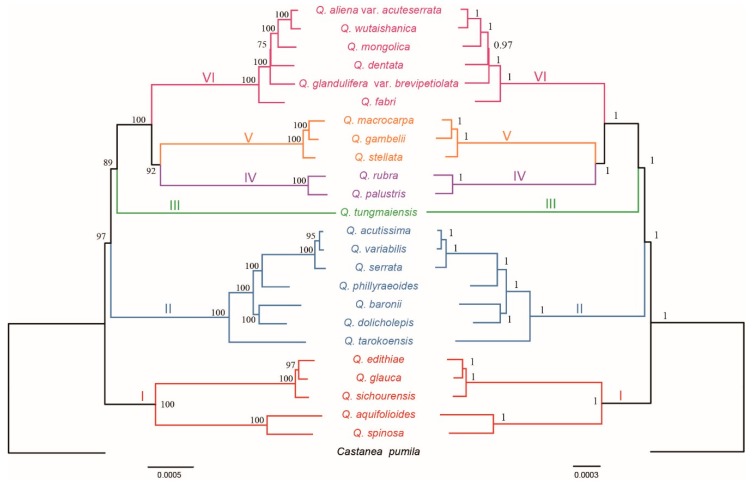
Phylogenetic tree inferred from the 25 chloroplast genomes. Left: Maximum likelihood tree with maximum likelihood (ML) bootstrap values; right: Bayesian tree with posterior probabilities.

The matrix of whole chloroplast genome sequences was used to reconstruct the *Quercus* phylogenetic tree (Figure 2). Both maximum likelihood and Bayesian analyses produced similar topologies for the 24 species and were highly branch supported. All the sampled *Quercus* species were clustered into one clade with 100% bootstrap value (BS) or Bayesian posterior probability (PP). However, backbone branch supports were relatively poor, as were some internal branches. Moreover, six major clades were identified in *Quercus* and the analyses obtained high support for all six of the nodes.

Clade I on the base of the tree (BS = 100% and PP = 1) comprised *Q. edithiae*, *Q. gambelii*, *Q. sichourensis*, *Q. aquifolioides*, and *Q. spinosa* being the earliest diverging lineages. Clade II (BS = 100% and PP = 1) contained seven species: *Q. acutissima*, *Q. variabilis*, *Q. serrata*, *Q. phillyraeoides*, *Q. dolicholepis*, *Q. baronii*, and *Q. tarokoensis*. Clade III only contained *Q. tungmaiensis*. *Q. rubra* and *Q. palustris* formed clade IV, which was identified as a sister to clade V with high support value (BS = 92% and PP = 1). Clade V included three species, *Q. macrocarpa*, *Q. glauca*, and *Q. stellata*. The last clade (BS = 100% and PP = 1) was made up of *Q. aliena* var. *acuteserrata*, *Q. wutaishanica*, *Q. mongolica*, *Q. fabri*, *Q. glandulifera* var. *brevipetiolata*, and *Q. dentata*. 

### 2.3. Analyses of the Standard DNA Barcodes

**Figure 3:**
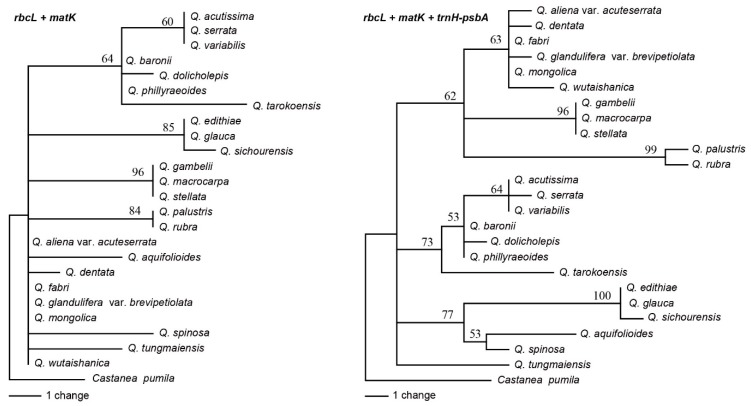
Neighbor joining trees for *Quercus* using *rbcL + matK*, *rbcL + matK*, and *trnH-psbA* combinations.

The *trnH-psbA* intergenic spacer region ranged from 412 bp to 474 bp with 27 variable sites, 16 informative sites, and nine indels of 3–20 bp within 574 aligned bp. A small 32 bp inversion occurred at 454 bp. *RbcL* and *matK* genes, both without indels, were 698 bp with eight variable and five informative sites, and 744 bp with 21 variable and 11 informative sites, respectively (Table 2). The mean interspecific genetic distances of the 24 oaks species with K2P were 0.0026 for *rbcL*, 0.0048 for *matK*, and 0.0125 for *trnH-psbA*. Based on the distance method, the universal DNA barcode had less discriminatory power; *rbcL*, *matK,* and *trnH-psbA* had only a 12.50%, 25.00%, and 37.50% success rate, respectively. With the two core DNA barcodes (*rbcL* and *matK*) combined, success was only 29.17%. Combined analyses of *rbcL*, *matK*, and *trnH-psbA* or *rbcL* and *matK* generated lower branch supported trees (Figure 3).

### 2.4. Analyses of Specific Barcodes

**Figure 4:**
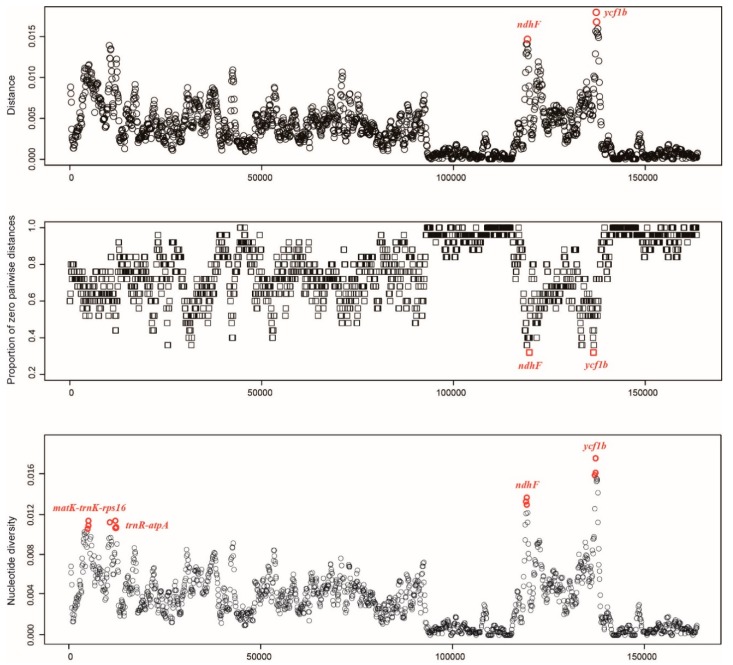
Specific DNA barcode development. (**A**) Mean distance of each window; (**B**) proportion of zero pairwise distances for each species; (**C**) nucleotide diversity (pi) of each window. Window length: 800 bp; Step size: 100 bp; X-axis: position of the midpoint of a window.

To identify closely related species, it is imperative to identify rapidly evolving markers. We used DNAsp and SPIDER to discover the variable mutation regions of the *Quercus* chloroplast genome (Figure 4). The nucleotide diversity (pi) value ranged from 0 to 0.01766 in the 800 bp window size, while the K2P-distance ranged from 0 to 0.0179. We found four relatively variable regions: *matK-trnK-rps16*, *trnR-atpA*, *ndhF,* and *ycf1b*. Two intergenic regions (*matK-trnK-rps16* and *trnR-atpA*) were located in the LSC region, and two coding regions (*ndhF* and *ycf1b*) in the SSC region. We designed new primers for four variable regions (Table S3).

**Figure 5:**
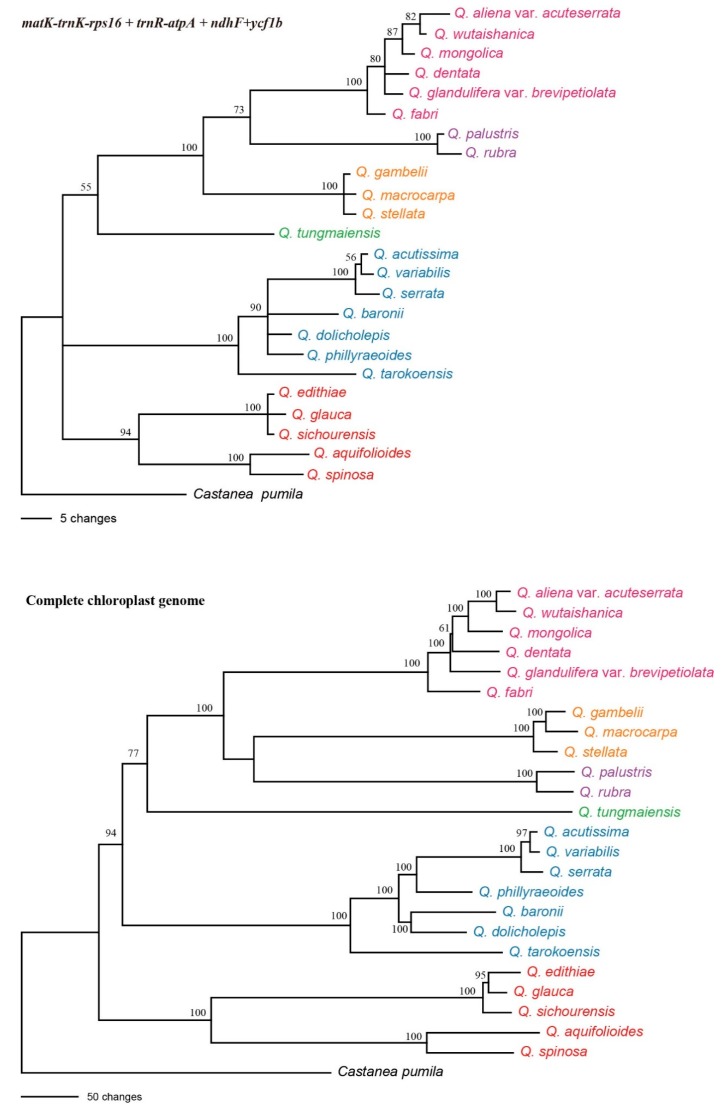
Neighbor joining tree for *Quercus* using the four highly variable markers and complete chloroplast genome data.

The *ycf1b* marker possessed the highest variability (5.33%), followed by the *ndhF* (4.82%), *trnR-atpA* (4.35%), and *matK-trnK-rps16* (4.02%) regions. Of the four variable makers, *ndhF* had the highest rate of correct identifications (83.33%), followed by *matK-trnK-rps16* (79.17%) and *ycf1b* (70.83%). Combining the four variable markers produced the most correct identifications (100%). The NJ tree-based method generated a graphical representation of the results and they were the same as those of the distance-based method (Figure 5).

### 2.5. Super-Barcode

The 24 *Quercus* chloroplast genomes were fully aligned, and an alignment matrix of 164,156 bp was obtained (Table 3). We identified 2778 variable sites (1.69%), including 1727 parsimony-informative sites (1.05%), in the total chloroplast genome. The average Pi value for the 24 *Quercus* chloroplast genomes was 0.00335. Among these regions, IR exhibited the least nucleotide diversity (0.00073) and SSC exhibited high divergence (0.00624).

To estimate the genetic divergence among *Quercus* chloroplast genomes, nucleotide substitutions and p-distance were calculated using MEGA. The overall sequence divergence estimated by p-distance among the 24 chloroplast genome sequences was only 0.0036. The number of nucleotide substitutions among the 24 species ranged from 14 to 734, and the p-distance ranged from 0.0001 to 0.0046. *Q. tungmaiensis* and *Q. serrata* had the largest sequence divergence. *Q. variabilis* had only 14 nucleotide substitutions with *Q. acutissima*.

The discriminatory power of the complete chloroplast genome as a DNA barcode was assessed using distance and tree-based methods. Compared to the standard DNA barcode or the four newly identified markers (specific barcodes), the complete chloroplast genome had the highest discriminatory power (Table 2 and Figure 5).

## 3. Discussion

Species delimitation remains one of the most controversial topics in biology. However, the accurate discrimination of material using only morphological characteristics is difficult. DNA barcoding is a widely used and effective tool that has enabled rapid and accurate identification of plant species since its development in 2003 [1]. Though DNA barcoding technology has developed significantly, no barcode can achieve the goal of sophisticated plant species identification [2]. In plants, the determination of a standardized barcode has been more complex. At present, increasing amounts of practical research tend to use chloroplast markers, such as *atpB-rbcL*, *atpF-H*, *matK*, *rbcL*, *psbK-I*, *rpoB*, *rpoC1*, *trnH-psbA*, and *trnL-F,* to identify species because of their relatively low evolutionary rates compared to those of nuclear loci and universal PCR primers [22,23,24,25]. The CBOL Working Group recently recommended a two-locus combination of *matK* + *rbcL* as the core plant barcode, with the recommendation to complement these using *trnH-psbA* and the ITS of the nuclear ribosomal DNA. However, because of the lower variability in standard DNA barcodes, discrimination power was low in plants [26]. In this study, the combination of *rbcL*, *matK*, and *trnH-psbA* had poor resolution (less than 50%) within *Quercus* (Table 2). Using the universal DNA barcode, the 12 Italian oak species revealed extremely low discrimination success (0%) [4]. Combined five chloroplast genome markers (*psbA-trnH, matK-trnK, ycf3-trnS, matK*, and *ycf1*), the species identification powers were only less than 20% [13]. Thus, there is an ongoing drive to develop additional oak barcodes. 

With sequencing method development, greater numbers of DNA sequences were easily acquired. Identification of specific barcodes was an effective strategy for barcoding complex groups. Most studies showed that chloroplast genome mutations were clustered into hotspots, and those hotspots were defined as DNA barcodes [27,28,29,30]. The strategy of searching the complete chloroplast genome has been successfully applied to *Oryza* [30], *Panax* [28], *Diospyros* [31], and *Dioscorea* [32]. By comparing 24 *Quercus* chloroplast genomes in the present study, we identified four oak-specific barcodes including *matK-trnK-rps16*, *trnR-atpA*, *ndhF,* and *ycf1b* (Figure 4). The *ycf1* gene was more variable than the *matK* and *rbcL* genes in most plant lineages, and recently has been the focus of a DNA barcoding and plant phylogeny study [14]. Furthermore, *ycf1* has previously provided a higher species resolution in *Quercus* [13,14]. The *ndhF* gene has been widely used in plant phylogeny and is considered a variable coding gene in the chloroplast genome [27,33,34,35]. *MatK-trnK-rps16* and *trnR-atpA* are two interspace regions less commonly used as DNA barcode. Combined with the four highly variable markers, all 24 *Quercus* species were successfully identified using the distance method (Table 2). 

Although the four specific barcodes had the highest discriminatory power, it was necessary to develop additional markers for *Quercus* because of its complex evolutionary history. With the advent of the next-generation DNA sequencing technologies, genomic data have extended the concept of DNA barcoding for species identification [6,8,36,37,38]. The DNA barcode has extended from gene or genes to the entire genome, and the extended DNA barcoding approach has been referred to as “ultra-barcoding” [39], “super-barcoding” [7], or “plant barcoding 2.0” [40]. Compared to the nuclear and mitochondrial genomes, the chloroplast genome is easily sequenced and may be the best-suited genome for plant species super-barcoding [36,41].

## 4. Materials and Methods

### 4.1. Taxon Sampling

The collection and GenBank accession information for taxa sampled in the present study are listed in Table 1 and Table S1. Ten species with previously sequenced chloroplast genomes used for analysis in this study are listed in Table S2. *Castanea pumila*, the sister group of *Quercus*, was used as the out-group.

### 4.2. DNA Extraction and Sequencing

We used an Illumina HiSeq X Ten platform to produce chloroplast genome sequences. *Quercus* species total DNA was extracted from silica-dried leaflets using the mCTAB protocol [42]. After extraction, total DNA was quantified with a Nanodrop 1000 Spectrophotometer. Fragmented samples of 350 bp were used to prepare paired-end libraries using a NEBNext® Ultra™DNA Library Prep Kit following the manufacturer’s protocol. Each library that passed the first quality control step was tested with an Agilent 2100 Bio-147 analyzer (Agilent Technologies, Santa Clara, CA, USA) to ensure the libraries had the required size distributions. Real-time quantitative PCR was carried out to precisely measure library concentrations to balance the amounts used in multiplexed reactions. Paired-end sequencing (2 × 150 bp) was conducted on an Illumina HiSeq X Ten platform. For each species, approximately 5 Gb of raw data were generated.

### 4.3. Genome Assembly and Genome Annotation

A five-step approach was used to assemble the chloroplast genome. First, raw sequence reads were filtered for high quality reads by removing duplicate reads, as well as adapter-contaminated reads and reads with more than five Ns using the NGS QC Tool Kit [43]. Second, the SPAdes 3.6.1 program [44] was used for de novo assemblies. Third, chloroplast genome sequence contigs were selected from the SPAdes software by performing a BLAST search using the *Quercus variabilis* chloroplast genome sequence as a reference. Fourth, the Sequencher 5.4.5 program (Gene Codes Corp., Ann Arbor, Michigan, USA) was used to merge the selected contigs. Finally, small gaps or ambiguous nucleotides were bridged with specific primers designed for PCR based on their flanking sequences by Sanger sequencing. The four junctional regions between the IRs and small single copy (SSC) and large single copy (LSC) regions in the chloroplast genome sequences were further checked by PCR amplification and Sanger sequencing with specific primers as previously described [45].

Chloroplast genome annotation was performed with Plann [46] using the *Quercus variabilis* reference sequence. The chloroplast genome map was drawn using OGdraw online [47].

### 4.4. Phylogenetic Analyses

Multiple sequence alignment was performed using MAFFT v7 [48]. We estimated phylogenetic trees on the nucleotide substitution matrix using maximum likelihood (ML) and Bayesian inference (BI). ML analyses were performed using RAxML v.8.1.24 [49].

The RAxML analyses included 1000 bootstrap replicates in addition to a search for the best-scoring ML tree. BI was conducted with Mrbayes v3.2 [50]. The Metropolis-coupled Markov chain Monte Carlo (MCMC) algorithm was run for 50,000,000 generations with one cold and three heated chains, starting with a random tree and sampling one tree every 2000 generations. The first 25% of the trees were discarded as burn-in, and the remaining trees were used to build a 50% majority-rule consensus tree. Stationarity was considered reached when the average standard deviation of split frequencies remained below 0.01.

### 4.5. Sequence Divergence and Hotspot Identification

We analyzed the aligned sequences and counted the sequence divergence among *Quercus* chloroplast genomes to evaluate *Quercus* species divergence. Variable, parsimony-informative base sites, p-distances across the complete chloroplast genomes, and LSC, SSC, and inverted repeat (IR) regions of the 14 taxa were calculated using MEGA 6.0 software [51].

We used two methods to identify the hypervariable chloroplast genome regions. The first (nucleotide variability) was conducted using DnaSP version 5.1 software with the sliding window method. The second (genetic distance) was conducted using the *slideAnalyses* function of SPIDER [52] version 1.2-0 software. This function extracts all passable windows of a chosen size in a DNA alignment and performs pairwise distance (K2P) analyses of each window. The proportion of zero pairwise distances for each species and mean distance were considered for the definition of hypervariable regions. The step size was set to 100 bp with an 800 bp window length.

### 4.6. DNA Barcoding Analysis

To access the effectiveness of marker discriminatory performance, we used two methods to assess the barcoding resolution. The distance method used the *nearNeighbour* function of SPIDER software [52]. The distance method was used to analyze the barcode performances of newly identified highly variable regions.

Tree building analyses provide a convenient and visualized method for evaluating discriminatory performance by calculating the proportion of monophyletic species. A neighbor joining (NJ) tree was constructed for each hypervariable marker and different marker combinations using PAUP* 4.0 software [53]. Relative support for the NJ tree branches was assessed via 200 bootstrap replicates.

## 5. Conclusions

In this study, we sequenced and compared the chloroplast genomes of 24 *Quercus* species. The structure, size, and gene content of the *Quercus* chloroplast genomes were found to be well conserved, and comparative analyses revealed low levels of sequence variability. Four higher variable regions were identified, which were suitable as DNA barcodes for *Quercus* species identification. We also evaluated the resolution of the complete chloroplast genome in phylogenetic reconstruction and species discrimination in *Quercus.* The complete chloroplast genome sequence data produced strongly supported and highly resolved phylogenies in this taxonomically complex group despite the extensive hybridization and introgression in *Quercus*. Compared to standard plant DNA barcodes and the specific barcodes, analyses of the complete chloroplast genome sequences improved species identification resolution.

## Figures and Tables

**Table 1:** Summary statistics for the assembly of 14 *Quercus* species chloroplast genomes.

Species	LSC	IR	SSC	Total Size (bp)	Number of Genes	Protein Coding Genes	tRNA	rRNA	Accession Number in Genbank
*Q. macrocarpa*	90594	25848	18946	161236	113	79	30	4	MK105459
*Q. gambelii*	90570	25848	18947	161213	113	79	30	4	MK105457
*Q. stellata*	90562	25848	18956	161214	113	79	30	4	MK105467
*Q. palustris*	90624	25852	18956	161284	113	79	30	4	MK105461
*Q. aliena* var. *acuteserrata*	90532	25837	18988	161194	113	79	30	4	MK105452
*Q. phillyraeoides*	90363	25866	19037	161132	113	79	30	4	MK105462
*Q. glandulifera* var. *brevipetiolata*	90534	25826	19038	161224	113	79	30	4	MK105458
*Q. wutaishanica*	90520	25825	19041	161211	113	79	30	4	MK105466
*Q. mongolica*	90504	25820	19047	161191	113	79	30	4	MK105460
*Q. dentata*	90593	25826	19055	161300	113	79	30	4	MK105453
*Q. fabri*	90557	25832	19064	161285	113	79	30	4	MK105456
*Q. serrata*	90447	25817	19065	161146	113	79	30	4	MK105464
*Q. variabilis*	90464	25817	19070	161168	113	79	30	4	MK105451
*Q. rubra*	90553	25870	19073	161366	113	79	30	4	MK105463

**Table 2:** The variability of the four new markers, chloroplast genome, and the universal chloroplast DNA barcodes in *Quercus.*

Markers	Length	Variable Sites		Information Sites		Discrimination Success (%) Based on Distance Method
		Numbers	%	Numbers	%	
*rbcL*	698	8	1.15%	5	0.72%	12.50%
*matK*	744	21	2.82%	11	1.48%	25.00%
*trnH-psbA*	574	27	4.70%	16	2.79%	37.50%
*rbcL + matK*	1442	29	2.01%	16	1.11%	29.17%
*rbcL + matK + trnH-psbA*	2016	56	2.78%	32	1.59%	50.00%
*matK-trnK-rps16*	2311	93	4.02%	59	2.55%	79.17%
*trnR-atpA*	1309	57	4.35%	35	2.67%	66.67%
*ndhF*	1536	74	4.82%	45	2.93%	83.33%
*ycf1b*	1765	94	5.33%	59	3.34%	70.83%
*ndhF+ycf1b*	3301	168	5.09%	104	3.15%	91.67%
*matK-trnK-rps16 + trnR-atpA + ndhF + ycf1b*	6921	318	4.59%	198	2.86%	100.00%

**Table 3:** Variable site analyses in *Quercus* chloroplast genomes.

	Number of Sites	Variable Sites		Information Sites		Nucleotide Diversity
		Numbers	%	Numbers	%	
LSC	92,888	2009	2.16%	1257	1.35%	0.0043
SSC	19,535	593	3.04%	368	1.88%	0.00624
IR	25,879	91	0.35%	54	0.21%	0.00073
Complete chloroplast genome	164,156	2778	1.69%	1727	1.05%	0.00335

## Supplementary Materials

Supplementary materials can be found at https://www.mdpi.com/1422-0067/20/23/5940/s1. Table S1. Sampling and assembly information for the 14 *Quercus* species; Table S2. A list of the 10 taxa sampled from GenBank in this study. Table S3. Primers for amplifying four highly variable loci.

## Abbreviations

LSC	Large single copy
SSC	Small single copy
IR	Inverted repeat
